# Effects of Environmental Enrichment on Doublecortin and BDNF Expression along the Dorso-Ventral Axis of the Dentate Gyrus

**DOI:** 10.3389/fnins.2017.00488

**Published:** 2017-09-15

**Authors:** Fabio Gualtieri, Catherine Brégère, Grace C. Laws, Elena A. Armstrong, Nicholas J. Wylie, Theo T. Moxham, Raphael Guzman, Timothy Boswell, Tom V. Smulders

**Affiliations:** ^1^Centre for Behaviour and Evolution, Institute of Neuroscience, Newcastle University Newcastle upon Tyne, United Kingdom; ^2^Brain Ischemia and Regeneration, Department of Biomedicine and Department of Neurosurgery, University Hospital Basel Basel, Switzerland; ^3^School of Psychology, Newcastle University Newcastle upon Tyne, United Kingdom; ^4^School of Natural and Environmental Sciences, Newcastle University Newcastle upon Tyne, United Kingdom

**Keywords:** hippocampus, neurogenesis, DCX, PROX1, BDNF, septo-temporal axis, enrichment, mouse

## Abstract

Adult hippocampal neurogenesis (AHN) in the dentate gyrus is known to respond to environmental enrichment, chronic stress, and many other factors. The function of AHN may vary across the septo-temporal axis of the hippocampus, as different subdivisions are responsible for different functions. The dorsal pole regulates cognitive-related behaviors, while the ventral pole mediates mood-related responses through the hypothalamic-pituitary-adrenal (HPA) axis. In this study, we investigate different methods of quantifying the effect of environmental enrichment on AHN in the dorsal and ventral parts of the dentate gyrus (dDG and vDG). To this purpose, 11-week-old female CD-1 mice were assigned for 8 days to one of two conditions: the Environmental Enrichment (E) group received (i) running wheels, (ii) larger cages, (iii) plastic tunnels, and (iv) bedding with male urine, while the Control (C) group received standard housing. Dorsal CA (*Cornu Ammonis*) and DG regions were larger in the E than the C animals. Distance run linearly predicted the volume of the dorsal hippocampus, as well as of the intermediate and ventral CA regions. In the dDG, the amount of Doublecortin (DCX) immunoreactivity was significantly higher in E than in C mice. Surprisingly, this pattern was the opposite in the vDG (C > E). Real-time PCR measurement of *Dcx* mRNA and DCX protein analysis using ELISA showed the same pattern. Brain Derived Neurotrophic Factor (BDNF) immunoreactivity and mRNA displayed no difference between E and C, suggesting that upregulation of DCX was not caused by changes in BDNF levels. BDNF levels were higher in vDG than in dDG, as measured by both methods. *Bdnf* expression in vDG correlated positively with the distance run by individual E mice. The similarity in the patterns of immunoreactivity, mRNA and protein for differential DCX expression and for BDNF distribution suggests that the latter two methods might be effective tools for more rapid quantification of AHN.

## Introduction

The hippocampus is a well-defined neuroanatomical structure in mammals, which includes two major subdivisions: the Dentate Gyrus (DG) and Ammon's horn (Latin: *Cornu Ammonis*—CA), the latter being subdivided into different subfields (CA1, CA2, and CA3; Andersen, 2007). Recently, increasing evidence points toward a functional division between the dorsal (*septal*) and ventral (*temporal*) poles of the hippocampus (Moser et al., 1993; Moser and Moser, 1998). The dorsal pole is proposed to be involved in the regulation of cognitive-related behaviors and long-term memory processing (Jessberger et al., 2009), while the ventral pole is proposed to mediate mood-related responses by means of the hypothalamic–pituitary–adrenal (HPA) axis (Bannerman et al., 2004). Acute activation of granule cells specifically in the dorsal DG (dDG) or in the ventral DG (vDG) differentially suppresses contextual learning or innate anxiety, respectively (Kheirbek et al., 2013). The dDG and the vDG, as well as the intermediate DG (iDG) differ in terms of gene expression (Fanselow and Dong, 2010) and connectivity (Thompson et al., 2008).

Adult hippocampal neurogenesis (AHN) occurs in the DG. Newly-born cells are generated in the sub-granular zone (SGZ) and mature into granule cells, the principal population of DG neurons (Kempermann et al., 2015; Nicola et al., 2015). AHN is sensitive to external factors: while physical exercise and enriched environment increase the number of new-born cells (Van Praag et al., 2002; Van Praag, 2009), chronic stress has the opposite effect (Mirescu and Gould, 2006). Chronic stress also alters the glucocorticoid negative feedback inhibition exerted by the hippocampus on the HPA axis (Smith and Vale, 2006). AHN differs along the dorso-ventral axis of the DG, in terms of baseline proliferation and maturation rates (Snyder et al., 2012). Training animals in a water maze causes higher activation of new neurons (as measured by c-Fos expression) in the ventral hippocampus, although there are more new neurons in the dorsal hippocampus (Snyder et al., 2009). AHN also responds differentially to external stimulation. For example, chronic stress affects neurogenesis in the vDG more than in the dDG (O'leary and Cryan, 2014). Consistent with this, the mechanism of many (although not all) antidepressant drugs to re-establish normal behavior in animal models of depression involves an increase in neurogenesis in the vDG (Santarelli et al., 2003; Boldrini et al., 2009; Tanti and Belzung, 2013a,b). This regional specificity of the AHN response does, however, depend on the exact conditions of the study (Wu and Hen, 2014).

There has been little investigation of the differential effect of environmental enrichment on the dorsal and ventral hippocampus. In rats, enriched environments reduced cytochrome c oxidase activity in brain regions related to the anxiety response, such as the ventral hippocampus, while its activity was not altered in the dorsal hippocampus (Sampedro-Piquero et al., 2013). In a similar study investigating the expression of synapsin I and glucocorticoid receptors (GR) in the dorsal and ventral hippocampus of standard and enriched rats, environmental enrichment increased synapsin I in the ventral CA3, and GR expression was increased in the vDG and the ventral subiculum (Sampedro-Piquero et al., 2014). Six weeks of environmental enrichment increases net hippocampal neurogenesis and the proportion of cells with more mature dendritic morphology along the entire dorsal-ventral dentate gyrus, although the effect is smaller in vDG than in dDG (Ramirez-Rodriguez et al., 2014). In that study, the number of proliferative cells is also increased in dDG but not in vDG. A similar effect was found in another set of studies, where 4–6 weeks of environmental enrichment preferentially increased AHN in the dDG, compared to vDG, while chronic stress mostly reduced AHN in the vDG (Tanti et al., 2012, 2013).

All these enrichment experiments included running wheels. It is well-known that voluntary exercise in running wheels by itself also increase AHN, both in terms of cell proliferation and maturation, and it can do so within 1 week (Jin et al., 2008). Another treatment that can increase neurogenesis, measured as number of maturing neurons, is exposure of female mice to male mouse urine (Mak et al., 2007), which is due to the presence of the protein Darcin (Hoffman et al., 2015). It is clear that 1 week of exposure to just voluntary exercise or to male mouse urine can increase the number of maturing new neurons in the DG. However, we do not know whether 1 week of stimulation is enough to affect dorsal and ventral hippocampus differentially. The first aim of our study is therefore to investigate whether 1 week of enrichment with a combination of stimuli (including male mouse urine and voluntary exercise) affects the dorsal hippocampus more than the ventral hippocampus.

A secondary aim of our study is to investigate whether molecular quantification of mRNA (through Real Time Polymerase Chain Reaction—RT-PCR) and of protein (using ELISA techniques) can speed up the measurements of AHN, while still obtaining similar results to the more traditional way of staining brain sections using immunohistochemistry, which is notoriously time consuming. We therefore, quantify AHN using immunohistochemical stains for Doublecortin (DCX), a microtubule-associated protein that is expressed specifically in migrating neuronal precursors and therefore commonly used as a marker of AHN (Couillard-Despres et al., 2005); and for Brain Derived Neurotrophic Factor (BDNF), which regulates neurogenesis, among other functions involved in brain plasticity (Castrén and Rantamäki, 2010). We also use RT-PCR to quantify the mRNA levels of these two genes, and ELISA to measure DCX protein levels in dorsal and ventral dentate gyrus of mice, after exposure to a week of environmental enrichment or to standard housing conditions. Because in immunohistological techniques, we quantify our measures relative to the size of the DG (or the total number of granule cells), we wanted to use a similar normalization procedure for the RT-PCR and the ELISA methods. Prospero homeobox protein 1 (PROX1) is a transcription factor constitutively expressed in all DG granule cells and not in any other hippocampal cell types (Iwano et al., 2012). We therefore also quantify the mRNA and protein levels of PROX1 in order to normalize DCX and BDNF expression relative to this measure of how many granule cells there are in the DG sample under investigation. We predict that the three methods will result in similar conclusions about the effect of 1 week of enrichment on AHN in dDG and vDG.

## Materials and methods

### Animals and experimental manipulation

**Figure 1:**
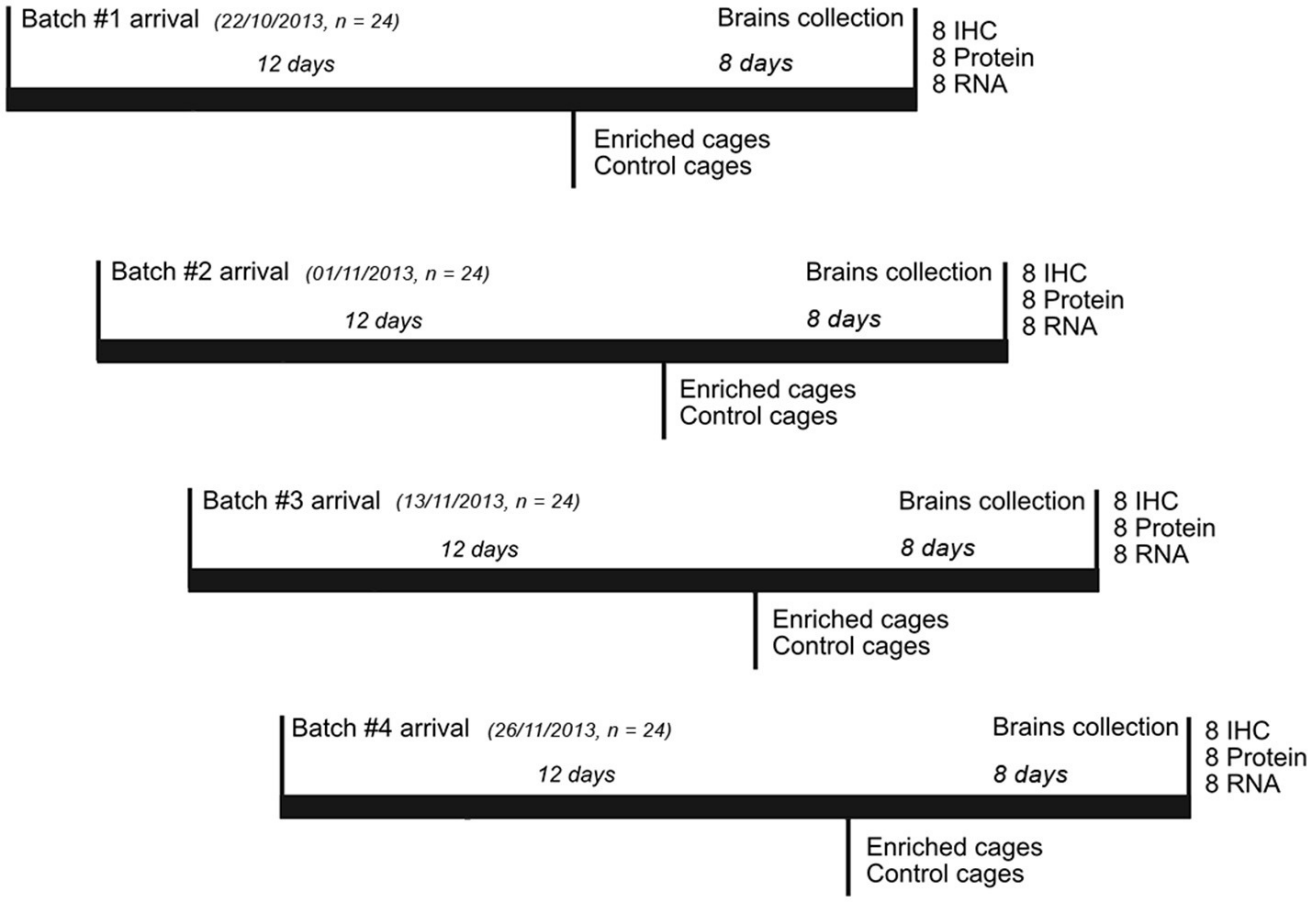
Experimental timeline. The experiment was run in 4 replicates of 24 animals each. Each batch of animals was allowed to settle into standard mouse housing in the Newcastle University mouse facility for 12 days. After this, half the cages were taken to a different room, and the animals transferred to the enriched environments (keeping the same social groups). The other half of the cages were kept in the standard mouse housing in the mouse facility. After a further 8 days, the animals were killed and brains were collected as described in the Section Materials and Methods.

We used 11-week-old female ICR mice (CD-1® Charles River, UK). The experiment was run in 4 batches of 24 animals each (for a total of 96 mice in the entire experiment). At the start of each batch, the animals were kept in standard housing cages, with three animals per cage, for the first 12 days. All were fed with normal chow and water *ad libitum* and kept on a 12:12 h light/dark cycle. Following this 12-day period, 4 cages of mice were randomly assigned to each of the two environmental conditions for the next 8 days: Enriched (E) or Control (C) (Figure 1). E mice were moved into a different room, separate from controls but keeping the same light/dark cycle.

**Figure 2:**
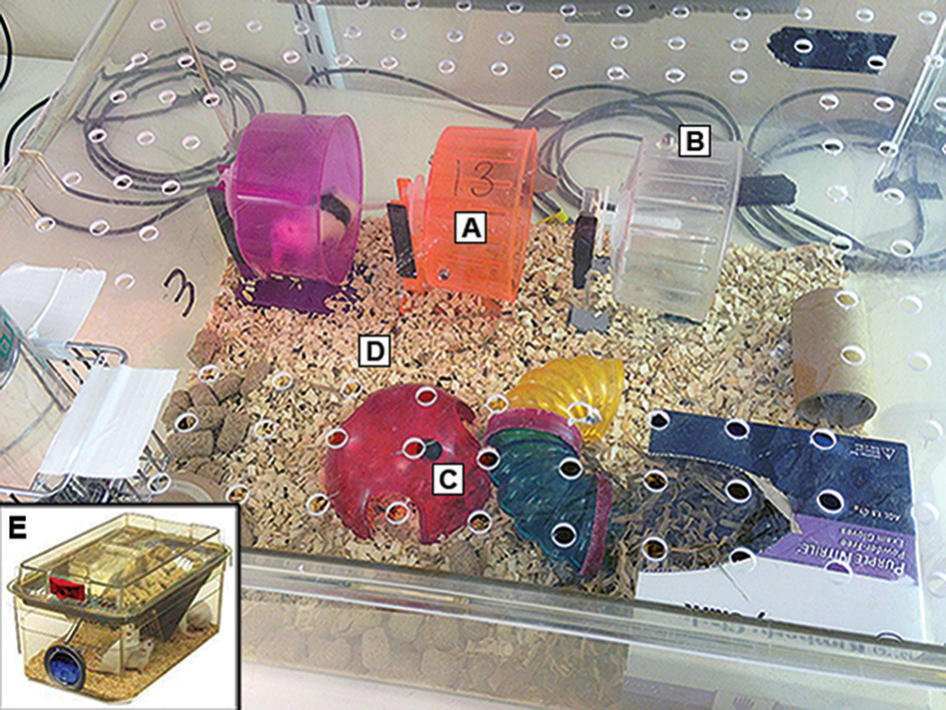
Enriched cage. Image of our enriched cage setting. For enriched animals we used a Perspex cage that was bigger than standard dimensions (48 × 38 × 21 cm) to facilitate activity and filled with **(A)** plastic and colored running wheels with **(B)** automatic rotation counters, **(C)** plastic tunnels and “igloos” as well as **(D)** soiled bedding from C57BL6 male mice. In order to avoid the stress of handling, animals were transferred using the “tunnel handling method” and animals were monitored during the dark phase with video cameras above the cage. The C condition **(E)** consisted of minimal housing in accordance with the UK 3Rs guidelines (nesting material, a wooden bite block and a cardboard tube), without any extra enrichment and with a standard size cage (39 × 20 × 16 cm) and minimal disturbance (bedding change every 3–4 days; mice transferred by being picked up by the tail).

The E condition (Figure 2) consisted of: (i) plastic and colored running wheels with automatic rotation counters (three wheels per cage), (ii) a cage bigger than standard dimensions (48 × 38 × 21 cm) to facilitate activity, (iii) plastic tunnels and “igloos,” and (iv) soiled bedding consisting of mixed dominant- and subordinate-male odors from C57BL6 male mice. We decided to use this bedding because male mouse urine contains high levels of Darcin, a major urinary protein that has been shown to increase hippocampal neurogenesis in female ICR strain mice (Mak et al., 2007; Roberts et al., 2010; Hoffman et al., 2015). In order to reduce the stress of handling, animals were transferred using the “tunnel handling method” (Hurst and West, 2010). Animals in the E group were also monitored during the dark phase with video cameras and red lighting, as well as spin counters to quantify the amount of wheel running performed by each mouse. For mouse identification in the video footage, E animals were marked with black hair dye according to one of the following designs: (i) no hair dye, (ii) one longitudinal black stripe on the back, (iii) two diagonal stripes on the back.

The C condition consisted of minimal housing in accordance with the UK 3Rs guidelines (nesting material, a wooden bite block, and a cardboard tube), without any extra enrichment and with a standard size cage (39 × 20 × 16 cm) and minimal disturbance (bedding change every 3–4 days; mice transferred by being picked up by the tail).

After 8 days in the respective treatments, brains were collected. We randomly chose an animal from either the E or C condition to start with. The next brain would then be from the other condition, and so on, alternating between animals from the two conditions (one cage at a time) until all brains had been collected. This was done to avoid possible confounds between time of day and treatment on the outcome measures. A total of 4 cages (2 E cages and 2 C cages) were processed per day. All the procedures were carried out under local (AWERB project ID: 343) and UK Home Office regulations, and mice were killed using a Schedule 1 method required in UK legislation.

### Estimation of physical activity

The distance each mouse ran in a wheel was quantified by analyzing video footage together with data recorded from the automated wheel counters in a systematic randomized manner. The first 3 min of every video-recorded hour from all cages was analyzed. Two observers analyzed alternate days of footage so that any bias in their recording of running times was distributed evenly across the cages and across time. From the video footage, we determined the proportion of running time that each mouse spent running in the wheel during those sample periods (a wheel was sometimes used by more than one mouse at once). For each experimental day, the number of seconds each mouse was observed to be running was divided by the total time that at least one mouse was observed running in the wheel in that cage. Then, the number of digitally recorded spins of the 3 wheels in each cage was totaled per day and allocated to the three mice according to the proportion of activity associated with each mouse (from the videos) in order to obtain an estimate of how many of the total daily wheel turns were made by each animal. Finally, the numbers of spins for each mouse were converted into the total distance traveled (km) using the circumference of the wheel, quantifying their differential levels of physical activity.

### Immunohistochemistry

One third of brains (*n* = 16 per treatment; one mouse per cage) were immersion fixed for 44–48 h in 4% paraformaldehyde in 0.01 M Phosphate Buffered Saline (PFA—PBS) at 4°C. They were then cryoprotected in a solution of 30% sucrose in 0.01 M PBS before being embedded in OCT (4,583, Electron Microscopy Sciences—USA). Coronal sections (40 μm) were cut on a cryostat (HM 550, Microm—Germany) and stored in cryoprotectant solution (30% glycerol, 30% ethylene glycol, 0.1M PBS). Serial sections, taken at 240 μm intervals, were then processed for immunohistochemistry.

For DCX staining, free-floating sections were washed in PBS at room temperature, endogenous peroxidase was inhibited for 30 min in 1% H_2_O_2_ in dH_2_O and tissue was permeabilized for 1 h in 0.01 M PBS containing 0.1% Triton X-100, 1 % bovine serum albumin and 2% normal horse serum. For BDNF staining, sections were processed in Tris buffered saline (TBS) instead of PBS because of the antibody requirements. Following 30 min of endogenous peroxidase inhibition in 1% H_2_O_2_ in dH_2_O, sections were washed three times with 0.05 M TBS pH 7.4 and then incubated for 1 h in blocking solution containing 0.3% Triton X-100, 5% bovine serum albumin and 5% normal goat serum in TBS. Samples were then incubated overnight (16 h) with a primary antibody raised in goat against DCX (Santa Cruz Biotechnology, sc-8066, RRID: AB_2088494) at 1:1,500 dilution or a primary polyclonal antibody raised in rabbit against BDNF (Abcam, ab72439, RRID: AB_1267795) at 1:1,000 dilution. The following day, after washing three times in PBS or TBS, sections were incubated for 2 h at room temperature with biotinylated horse anti-goat IgG (Vector Laboratories, BA-9500, RRID: AB_2336123) or biotinylated goat anti-rabbit IgG (Vector Laboratories, BA-1000, RRID: AB_2313606), respectively. Sections were then incubated at room temperature for 1 h with horseradish peroxidase streptavidin (Vector Laboratories, SA-5004, RRID: AB_2336509) and stained using the avidin–biotin complex indirect technique with SIGMAFAST 3,3′-Diaminobenzidine tablets (Sigma Aldrich, D4293) as chromogen (Gualtieri et al., 2012). Brain samples were then rinsed in water, dried on a pre-warmed hotplate at 37°C and coverslipped with Eukitt (Sigma Aldrich, 03989) for image analysis.

### Image analysis

#### Dorso-ventral discrimination

For every animal, the DG in four to six serial sections (at 480 μm intervals) was captured at 20X magnification using a Leica DMLB microscope connected to a video camera (Optronics Microfire Digital Camera, USA). Images acquired were processed for DG analysis with ImageJ (NIH, USA). To investigate differences between dorsal and ventral regions we initially subdivided the whole DG into three different parts according to Fanselow and Dong (2010). The three regions, namely vDG, iDG, and dDG, were defined with Brain Explorer 2 software (Allen Brain Institute, USA). Briefly, on the sagittal plane images we identified where dorsal and ventral parts merge and the CA3 pyramidal neurons cluster together, appearing as a X-shaped pyramidal pool (Figure S1A) that is visible on one particular “re-sliced” sagittal plane of the Allen Reference Atlas (≈2.494 mm from the midline). By translating these borders on the coronal plane (Figure S1B) and moving the coronal plane toward the rostral pole (Figures S1C–E) or the caudal pole (Figure S1F) we were able to determine the anatomical landmarks of the iDG, the region that was excluded. The dorsal limit of iDG was represented by the pre commissural nucleus (PRC) while the ventral limit was represented by both the interstitial nucleus of Cajal (INC) and the nucleus of Darkschewitz (ND). The identification of these landmarks throughout our sections (Figures S1C′–F′) allowed us to discriminate the limits of the three sub-regions. After slicing the brain according to the mouse stereotaxic atlas (Paxinos and Watson, 2007) more sections contained the dDG than the vDG owing to the cutting angle. However, since we normalized for the amount of DG GCL that was sampled, this is controlled for in our analyses.

#### Doublecortin quantification

**Figure 3:**
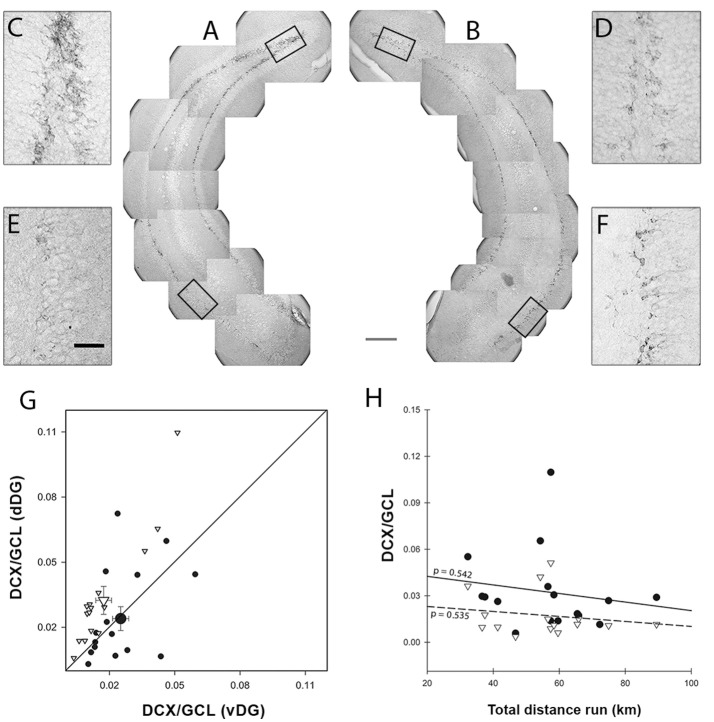
DCX Morphological analysis. Image montage of representative dentate gyrus (DG) coronal sections of Enriched **(A)** and Control **(B)** animals (14-week-old CD-1 female mice) showing different amounts of DCX staining in dorsal and ventral DG (dDG and vDG, respectively). The inset magnification shows that the amount of staining in E-dDG **(C)** is higher than in C-dDG **(D)** while conversely it is lower in E-vDG **(E)** compared to C-vDG **(F)**. *Scale bars*: gray = 225 μm, black = 50 μm. Panel **(G)** shows the amount of dorsal DCX staining plotted against the amount of ventral DCX staining. In the Enriched animals, the proportion of dorsal DCX^+^ pixels is higher than ventral ones [points above the diagonal; EdDG vs. EvDG $\chi_{\left(1\right)}^{2}$ = 14.835 *p* < 0.001], while in the Control animals, dorsal and ventral are equivalent. Black dots = Control, white inverted triangles = Enriched. Bigger symbols represent means with bi-dimensional bars representing SEM. **(H)** The effect of distance run, plotted against DCX/GCL showed no significant effects or interactions. Black dots = dDG, white inverted triangles = vDG, continuous line = dDG regression line, dashed line = vDG regression line.

In the serial sections through the hippocampus, we quantified the relative area of the dentate gyrus that was DCX^+^ (Ayzenberg et al., 2015). We considered this the most appropriate measure to relate to the mRNA and protein quantification, as they also integrate the number of DCX-expressing cells with the size of those cells. The person performing the quantification was blind to the treatment group to which the animals belonged. Because staining intensity and background can vary among sections or staining batches, we applied a customized gray value threshold for every brain section to determine which pixels represented DCX-positive cells. To define this threshold, the GCL region was outlined (Figure S2A) and every picture frame from the same section was converted from RGB (Figure S2C_1_) to the 8-bit grayscale image (Figure S2C_2_; Figure 3).

Following this transformation, for each picture of the composite image, we outlined a region within the GCL with no DCX staining (triangle, Figure S2C_3_). We used the ImageJ histogram function (Figure S2C_4_) to obtain the SD of the gray scale values for each image. The threshold used as minimum gray value representing DCX^+^ staining was calculated as follows:

(1) $$\begin{array}{l} Threshold=Mean-2SD \end{array}$$

All the composite images were then elaborated in the same way and the threshold applied to every frame in a montage (Figure S2B, sequential 1–8), thus resulting in the total DCX-positive area (in μm^2^) for each image. We also outlined and measured the total area of the granule cell layer (GCL) representing the whole GC population in the DG. For both the DCX and GCL areas, values from every picture frame were summed to have a total per section (separately for dDG and vDG). These values were then added together to obtain total values per animal (left and right hemispheres were combined). These two measurements (in μm^2^) were then used as follows to obtain a normalized value for the amount of DCX in vDG and dDG in both E and C groups:

(2) $$\begin{array}{l} DCX\;\left(normalized\right)\;=\frac{DCX\;area}{GCL\;area} \end{array}$$

#### BDNF quantification

**Figure 4:**
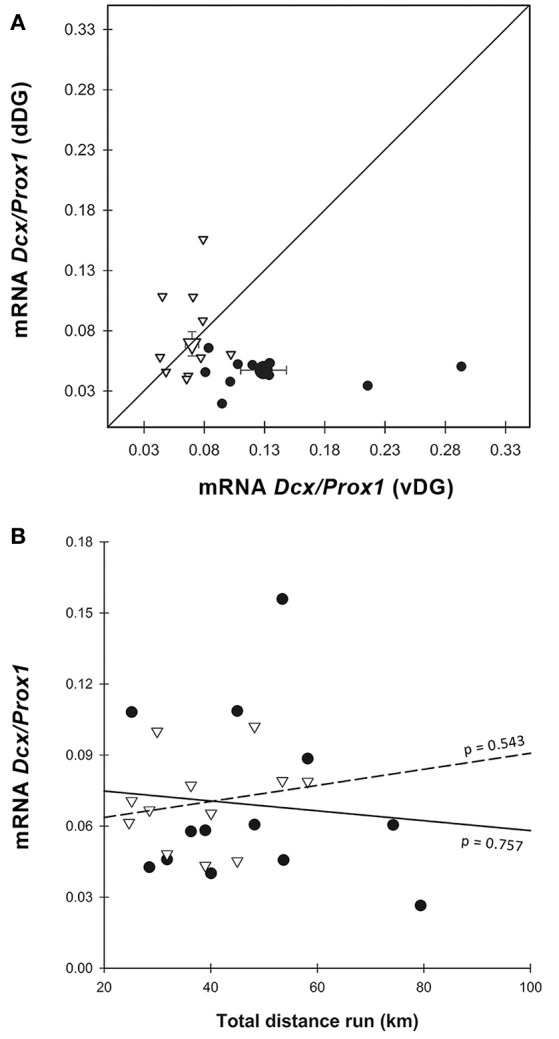
*Dcx* gene expression. **(A)** When normalized on *Prox1, Dcx* was increased in E-dDG compared to C-dDG (GEE, *p* = 0.033) but decreased in E-vDG compared to C-vDG (GEE, *p* = 0.002). Both C-vDG and E-vDG were significantly different from C-dDG (GEE, *p* < 0.001) and E-dDG was different from C-vDG (GEE, *p* = 0.004). Note that, like in Figure 3G, the points of the enriched animals are found to the left and above those of the control animals. Legend for **(A)** as in Figure 3G. **(B)** The effect of distance run, plotted against *Dcx* mRNA showed no significant effects. Legend for **(B)** as in Figure 3H.

The person performing the quantification was blind to the treatment group to which the animals belonged. For BDNF analysis, four to six sections per animal were captured at 10X magnification using the same set-up and converted to 8-bit gray scale as before. Unlike DCX expression, BDNF staining is uniform across the entire DG, so we cannot measure the proportion of the surface area represented by BDNF^+^ staining. Instead, we decided to quantify the darkness of the staining itself as a proxy of the density of BDNF molecules in the tissue. To quantify BDNF expression, four rectangular sample regions of interest were outlined in each sampling location: one each in the suprapyramidal and infrapyramidal blades of the DG, the hilus and the mossy fibers (Figure 4). This was done in four dorsal and four ventral sampling regions for each animal. The relative optical density (ROD) of each region of interest was measured using ImageJ according to the software calibration toolset (please refer to “ImageJ—Tutorials and Examples—Optical Density Calibration” web page that can be found at the following link: https://imagej.nih.gov/ij/docs/examples/calibration/index.html). The average ROD of the corpus callosum was also quantified for each section and then subtracted from the DG ROD values to control for background gray values (Neeper et al., 1996).

#### Volume quantification

In order to estimate hippocampal volume, we divided it in three different subregions according to Fanselow and Dong (2010), namely the dorsal hippocampus (dHip), intermediate hippocampus (iHip), and ventral hippocampus (vHip; Figure S1). Again the person performing the quantification was blind to the treatment group to which the animals belonged. Volumes were calculated from 2 sets of slides, each containing roughly half of the brains. Fifteen brains (8 control, 7 enriched) were stained with cresyl violet. The other 17 (8 control, 9 enriched) were stained with the BDNF antibody. We outlined the different hippocampal fields separately, outlining dorsal hippocampus (DG and CA) every 480 μm, and intermediate and ventral hippocampus (DG and CA) every 240 μm. Volumes were calculated by multiplying the area of the outline by the distance to the next section (or by 240 for the last section) and then adding up all these volumes. Volumes are expressed in mm^3^. We again combined left and right hemispheres, as we were not always able to identify left and right hemisphere in all the sections, because tissue damage sometimes obscured the hole we introduced into one hemisphere as a hemisphere identifier. Because the same boundaries could not be identified in antibody stained (background-stained) and cresyl violet stained tissue, the estimate of the dorsal (but not intermediate or ventral) hippocampus volume was higher in antibody stained tissue compared to cresyl violet stained tissue [counterstained ^*^ longitudinal axis interaction: *F*_(2, 26)_ = 7.99, *p* = 0.002], so we controlled for staining method by including it as a categorical variable in the analysis.

### Molecular quantification

For the remaining 64 mice (32 per group), the DGs were dissected from each brain hemisphere using the methods described by Hagihara et al. (2009). Briefly, brains were dissected from the skull and placed into ice-cold PBS in a Petri dish. They were then divided along the longitudinal fissure with a scalpel and the regions posterior to lambda (midbrain, hindbrain, and cerebellum) were cut off. The diencephalon (thalamus and hypothalamus) was removed under a stereomicroscope uncovering the medial side of the hippocampus, from which the DG was dissected out. The isolated DG was then divided into two equal parts (dDG and vDG), placed in a sample tube and fresh-frozen in dry ice. Left and right hemispheres were combined for each animal.

#### Quantitative RT-PCR

For 32 of these brains (16 per group; one from each cage), RNA was extracted using TRI Reagent (T9424, Sigma-Aldrich, UK) and Lysing Matrix D tubes in a FastPrep Instrument (MP Biomedicals, UK). Total RNA was reverse-transcribed using a QuantiTect reverse transcription kit (Qiagen) that incorporates a DNase treatment step. We performed real-time PCR experiments using the Rotor-Disc® 100 on a Rotor-Gene® Q thermal cycler (Qiagen, Germany) using QuantiTect mouse primer mixes (Qiagen). The primer sequences are proprietary; the primer mixes used were for *Dcx* (Qiagen QT02521155), *Bdnf* (QT00097118), and *Prox1* (QT01070615). Standards were produced by gel purification of PCR products using a QiaQuick gel extraction kit (Qiagen) and their concentration was measured on a NanoDrop spectrophotometer (Thermo Fisher Scientific). Serial dilutions of standards were made for real-time PCR quantification. Amplifications were performed using a SensiFAST SYBR no-ROX kit (Bioline) in 20 μl volume reactions containing the QuantiTect forward and reverse primer mix at the manufacturer's recommended dilution to produce the optimal concentration, and 2 μl cDNA. All of the genes were run in singlicate with all samples together in one run. All runs contained a no-template control together with the diluted standards. The following cycling program was used: 95°C for 5 min followed by 40 cycles of 95°C for 15 s and 60°C for 10 s. A melting curve analysis was performed to confirm specificity of the PCR reaction. As expected, each melting curve revealed a single peak, corresponding to the desired specific amplification product. Primer efficiency ranged from 98–104%.

In the dentate gyrus, amounts of *Dcx* and *Bdnf* mRNA were expressed relative to levels of *Prox1*. This gene is expressed predominantly in granule cells within the DG during development (Lavado et al., 2010) and remains expressed throughout the lifetime of these neurons. *Prox1* allowed us to normalize our neurogenesis related genes to the total population of granule cells, just as we expressed the morphological analysis as a proportion of DCX positive area relative to the total GCL area.

#### ELISA tissue processing

The remaining 32 animals' dorsal and ventral dentate gyrus samples were homogenized using a polypropylene pestle fitted onto a motorized hand-held homogenizer in 10 volumes (w/v) of ice cold RIPA buffer (10 mM Tris/HCl pH7.4, 100 mM NaCl, 1 mM EDTA, 1 mM EGTA, 1% Triton X-100, 10% glycerol, 0.1% SDS, 0.5% sodium deoxycholate) freshly supplemented with 50 mM sodium fluoride, 1 mM activated sodium orthovanadate, phosphatase inhibitor cocktail A (J65354, Alfa Aesar), and protease inhibitor cocktail (11836170001, Complete Mini EDTA-free, Roche). Samples were then centrifuged at 14,000 g for 20 min at 4°C. Supernatants were immediately collected and stored at −80°C until further use. Protein concentration was determined using the Bradford assay.

The sandwich immunoassay for DCX was performed on the Meso Scale Discovery instrument as described in Kremer et al. (2013), with minor modifications. A 96-well streptavidin plate (Mesoscale Cat No L15SA-1) was blocked for 2 h at room temperature in Tris-buffered saline (TBS, 50 mM Tris/HCl, pH 7.4, 60 mM NaCl) supplemented with 0.1% Tween-20 and 5% bovine serum albumin (BSA). After 3 washes in TBS/0.1% Tween-20, the plate was coated overnight at 4°C with the capture antibody, i.e., 10 nM biotinylated mouse anti-DCX antibody (mAb83) diluted in assay buffer (TBS with 0.1% Tween-20 and 0.5 % BSA). Fifty microliters of standard (recombinant human DCX, 0.61-10,000 pg/ml) or samples, diluted in the assay buffer were incubated together with an MSD SULFO tag labeled mouse anti-DCX antibody (mAb49, 2 nM) for 4 h at room temperature with shaking at 600 rpm. Standards, samples and blanks were analyzed in duplicates. The plate was washed again 3 times, and then read on a MSD Sector Imager 6000 plate reader after addition of read buffer (MSD). Mouse anti-DCX antibodies (mAb83 and mAb49) and recombinant human DCX were a generous gift from Thomas Kremer (Roche). An enzyme-linked immunosorbent assay kit (MyBioSource Inc. San Diego, California, USA) was used to quantify PROX1 according to the manufacturer's instructions, using 10 μl of undiluted dentate gyrus tissue sample. The person performing the assay was blind to the treatment group to which the animals belonged.

### Statistical analysis

All datasets were initially processed with the Inter Quartile Range rule with the multiplier set at 2.2 (Hoaglin et al., 1986) in order to detect outliers. Unless otherwise specified, all the data were analyzed using the Generalized Estimating Equations (GEE) model in SPSS 22 (IBM, USA) and all descriptive statistics are expressed as mean ± SEM. The GEE model in SPSS can analyze the same kind of data as could be analyzed with a traditional repeated-measures ANOVA, but is able to handle missing data in a more sophisticated manner. We therefore preferred using GEE over traditional GLM or ANOVA models. Data from two or more regions of the same animal were treated as repeated measures (within subject variables). *p*-values are based on the Wald's χ^2^ test statistics and parameters were estimated by a maximum likelihood approach. Wheel-running distance was inserted as a co-variate in the analysis when investigating the effect of wheel running on hippocampal parameters. For all analyses we used the least significant differences *post-hoc* correction for multiple testing and *p* < 0.05 was considered statistically significant.

## Results

### Doublecortin

#### Morphological quantification

Significantly more DCX immunoreactivity was detected in dDG than in vDG [$\chi_{\left(1\right)}^{2}$ = 5.370, *p* = 0.020] and this effect was more pronounced in the experimental group [Figures 3A–G; enrichment*region: $\chi_{\left(1\right)}^{2}$ = 14.838, *p* < 0.001]. The two regions responded differently to the enriched environment, presenting a 1.4-fold increase in DCX immunoreactivity in dDG and a 1.4-fold decrease in vDG compared to the same regions in the control animals. When we entered the distance run by enriched animals as a covariate in the GEE to correlate with DCX immunoreactivity, we found that this had no effect [Figure 3H; distance: $\chi_{\left(1\right)}^{2}$ = 1.792, *p* = 0.181] in either dDG (*r*^2^ = 0.027) or vDG [*r*^2^ = 0.030; region^*^distance: $\chi_{\left(1\right)}^{2}$ = 0.058, *p* = 0.810].

#### Gene expression

In order to estimate the relative amount of neurogenesis to granule cells we normalized the expression of *Dcx* to *Prox1* (Figure 4A). This ratio showed higher expression in vDG than in dDG [$\chi_{\left(1\right)}^{2}$ = 14.188, *p* < 0.001] across all animals. The pattern was significantly different in E compared to C [enrichment*region: $\chi_{\left(1\right)}^{2}$ = 14.326, *p* < 0.001] but there was no significant main effect of enrichment [$\chi_{\left(1\right)}^{2}$ = 2.837, *p* = 0.092]. In dDG, enriched animals expressed higher levels of *Dcx* than controls (*p* = 0.030). In contrast, in vDG the expression was markedly lower in enriched animals than controls (*p* = 0.002). Using running distance as a covariate in the GEE (Figure 4B), we found no effect of distance run on the amount of *Dcx*/*Prox1* in either dorsal (*r*^2^ = 0.009) or ventral (*r*^2^ = 0.038) dentate gyrus [distance: $\chi_{\left(1\right)}^{2}$ = 0.016, *p* = 0.900; region^*^distance: $\chi_{\left(1\right)}^{2}$ = 0.466, *p* = 0.495].

#### Protein expression

**Figure 5:**
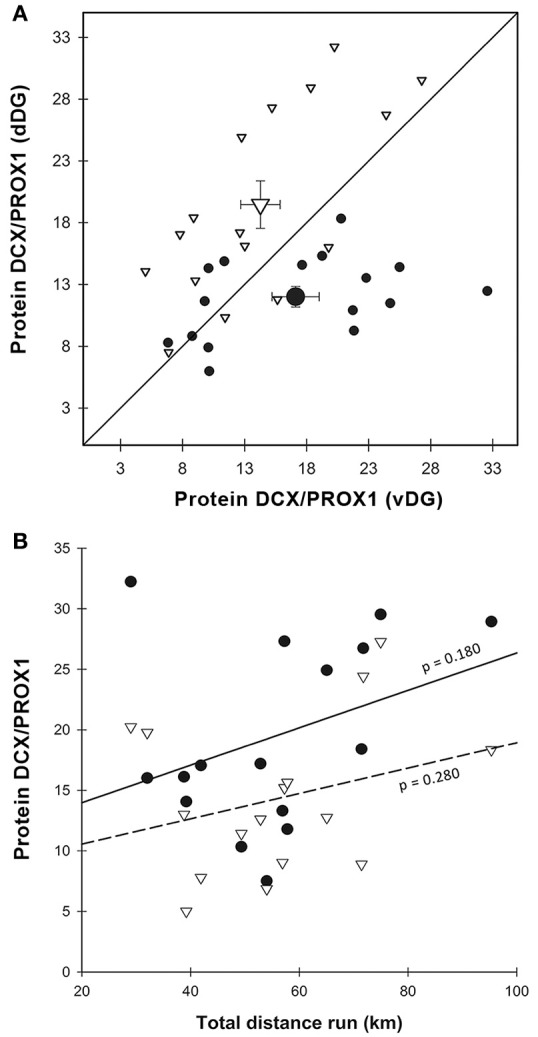
DCX protein expression. **(A)** DCX protein content normalized over PROX1 was significantly higher in E-dDG than in C-dDG and E-vDG (χ^2^ = 22.938, *p* < 0.001), while in C-vDG it was higher than in C-dDG (*p* = 0.003). As in Figures 3G, 4A, the data points representing the enriched group lie to the left and above those representing the control animals. Legend for **(A)** as in Figure 3G. **(B)** The effect of distance run, plotted against DCX protein expression showed no significant effects. Legend for **(B)** as in Figure 3H.

DCX protein content was also normalized to PROX1. This ratio followed the same pattern we found both with morphological analysis and real-time PCR (Figure 5A). There was a strong interaction between region and enrichment [$\chi_{\left(1\right)}^{2}$ = 22.938, *p* < 0.001], but no main effect of either region [$\chi_{\left(1\right)}^{2}$ = 0.004, *p* = 0.953] or enrichment [$\chi_{\left(1\right)}^{2}$ = 1.376, *p* = 0.241]. In particular, we found a significant (*p* < 0.001) 1.6-fold increase of DCX content in dDG in enriched, compared to control animals, while in vDG the pattern was completely reversed with enriched animals displaying a significant 1.2-fold decrease (*p* < 0.001) in DCX content compared to controls. Using running distance as a co-variate in the GEE, we found no effect of distance run on the amount of DCX/PROX1 in either dorsal (*r*^2^ = 0.125) or ventral (*r*^2^ = 0.083) dentate gyrus [$\chi_{\left(1\right)}^{2}$ = 1.878, *p* = 0.171, Figure 5B].

### Brain-derived neurotrophic factor

#### Morphological quantification

**Figure 6:**
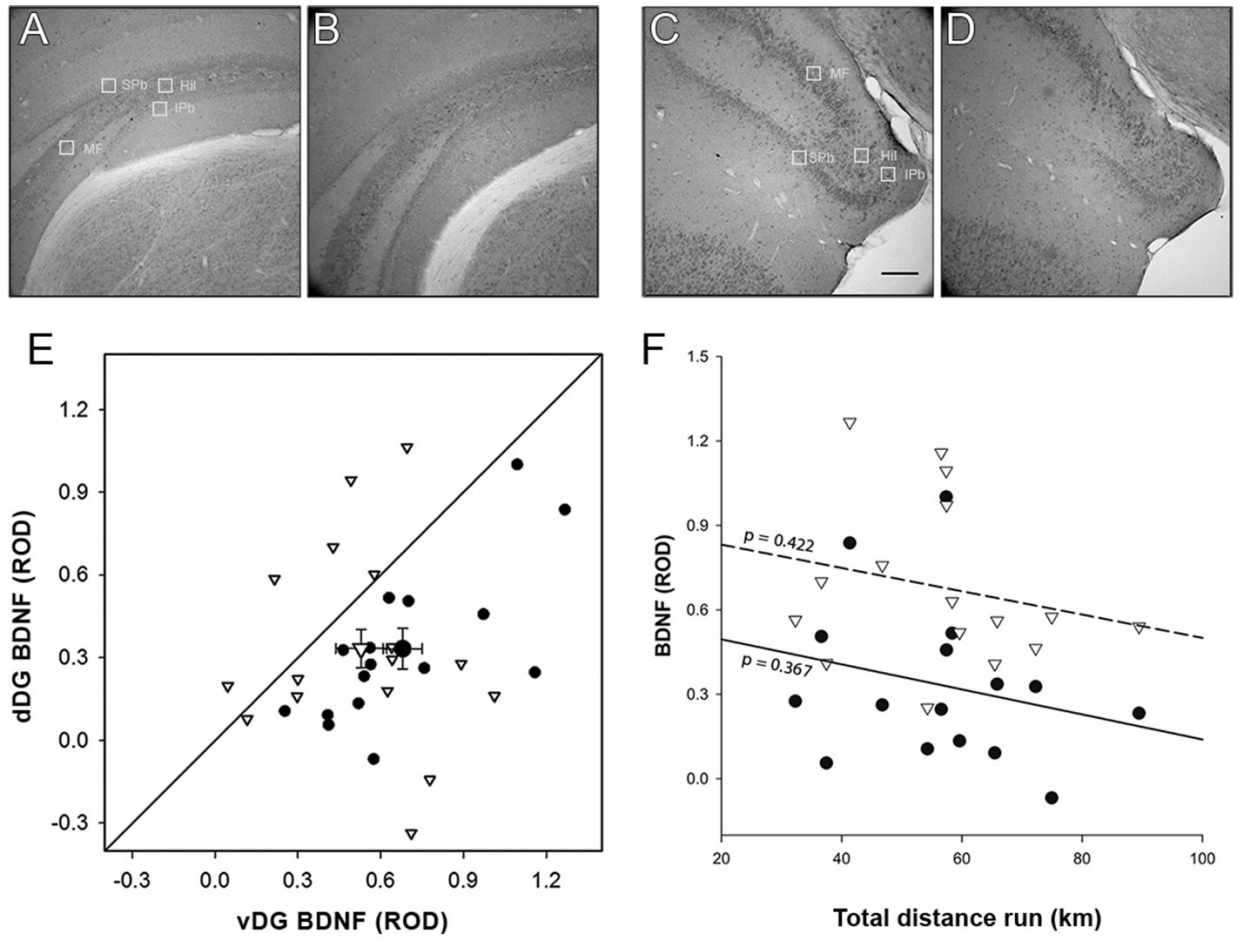
BDNF Morphological analysis. Optical density of BDNF immunohistochemistry was significantly higher [$\chi_{\left(1\right)}^{2}$ = 18.180, *p* < 0.001] in both EvDG **(C)** and CvDG **(D)** compared to their dorsal counterparts (**A,B**, respectively) *Scale bar*: 70μm. The great variability found in E animals gave no significant difference from C animals which were mostly distributed below the identity line in plot **(E)**. Enriched environment did not have an effect on BDNF immunoreactivity [$\chi_{\left(1\right)}^{2}$ = 0.802, *p* = 0.371]. No significant interaction was found between experimental treatment and region [enrichment*region: $\chi_{\left(1\right)}^{2}$ = 1.398, *p* = 0.237]. Legend for **(E)** as in Figure 3G. **(F)** The effect of distance run, plotted against BDNF ROD showed no significant effects. Legend for **(F)** as in Figure 3H.

Left and right hippocampus were summed together in order to have a total expression for each experimental category (Figures 6A–E). BDNF expression varied between the sampling sites in the DG, being lowest in the hilus, and significantly lower in the infrapyramidal blade than in the suprapyramidal blade and mossy fibers [$\chi_{\left(3\right)}^{2}$ = 63.466, *p* < 0.001]. Enriched environment did not have an effect on BDNF immunoreactivity [$\chi_{\left(1\right)}^{2}$ = 0.801, *p* = 0.371] but a strong difference along the dorso-ventral axis was detected: BDNF expression was more abundant in vDG than dDG [$\chi_{\left(1\right)}^{2}$ = 18.188, *p* < 0.001]. No significant interaction was found between experimental treatment and sampling site [enrichment^*^site: $\chi_{\left(3\right)}^{2}$ = 0.957, *p* = 0.812] or the dorso-ventral axis [enrichment^*^region: $\chi_{\left(1\right)}^{2}$ = 1.408, *p* = 0.235], but a significant interaction between sampling site and dorso-ventral axis was present [site^*^region: $\chi_{\left(1\right)}^{2}$ = 18.741, *p* < 0.001].

When we entered the distance run by enriched animals as a covariate in the GEE to correlate with the BDNF ROD (Figure 6F), we found that this had no effect [$\chi_{\left(1\right)}^{2}$ = 2.319, *p* = 0.128], in either vDG (*r*^2^ = 0.047) or dDG [*r*^2^ = 0.059; region^*^distance: $\chi_{\left(1\right)}^{2}$ = 3.399, *p* = 0.065]. Even though a significant three-way interaction was present [site^*^region^*^distance: $\chi_{\left(3\right)}^{2}$ = 13.641, *p* = 0.003], analysis of all subdivisions separately did not reveal any site/subregion in which the distance run significantly predicted the amount of BDNF.

#### Gene expression

**Figure 7:**
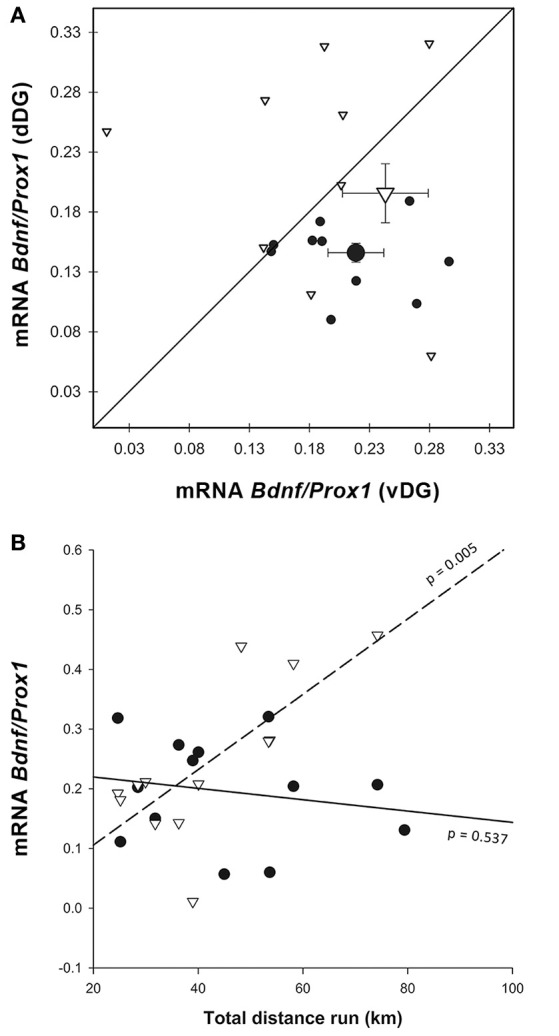
*Bdnf* gene expression. **(A)** Most of the Enriched animals showed an increase in dorsal BDNF compared to controls, but some show a decrease, causing larger variability in that group and no significant difference from controls. Legend for **(A)** as in Figure 3G. **(B)** The effect of distance run, plotted against *Bdnf* mRNA showed a significant interaction, where *Bdnf*/*Prox1* is correlated with running distance in vDG [*r*^2^ = 0.535, region^*^distance $\chi_{\left(1\right)}^{2}$ = 35.705, *p* < 0.001], but not dDG. Legend for **(B)** as in Figure 3H.

Similar to the immunohistochemistry result, *Bdnf* expression normalized on *Prox1* (Figure 7A) was higher in vDG than in dDG [$\chi_{\left(1\right)}^{2}$ = 6.056, *p* = 0.014], but no differences were found due to enrichment [enrichment: $\chi_{\left(1\right)}^{2}$ = 2.447, *p* = 0.118; enrichment*region: $\chi_{\left(1\right)}^{2}$ = 0.193, *p* = 0.661]. When we used distance as a covariate in the GEE (Figure 7B), we found that mice which ran longer distances expressed more *Bdnf* /*Prox1* in ventral (*r*^2^ = 0.535) but not in dorsal DG [*r*^2^ = 0.036; region^*^distance: $\chi_{\left(1\right)}^{2}$ = 35.705, *p* < 0.001].

### Hippocampal volume

**Figure 8:**
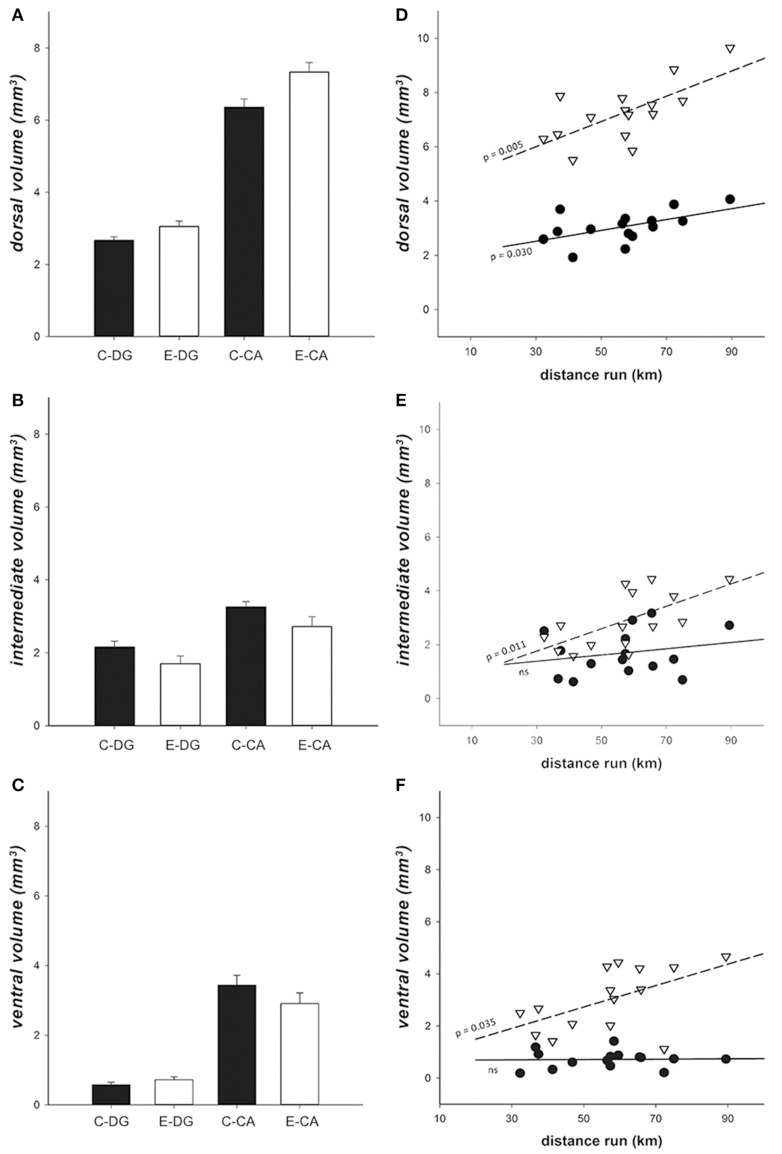
Hippocampal volume. **(A–C)** Volume comparison showed that enrichment increased dorsal hippocampal volume, but slightly decreased volume of intermediate and ventral hippocampus. Black bars = Controls, white bars = Enriched. **(D–F)** In enriched animals, individual volume variation in dorsal **(D)**, intermediate **(E)**, and ventral **(F)** CA and DG regions in relation to the distance run. There was a significant interaction between distance run, DG vs. CA, and the three sub-regions [$\chi_{\left(2\right)}^{2}$ = 84.049, *p* < 0.001]. Particularly, in dorsal hippocampus **(D)** both DG (*r*^2^ = 0.302, *p* = 0.030) and CA (*r*^2^ = 0.464, *p* = 0.005) showed a linear correlation with running. In iHip **(E)** and in vHip **(F)** only CA showed a correlation between the distance run and the hippocampal volume (iCA: *r*^2^ = 0.402, *p* = 0.011; vCA: *r*^2^ = 0.301, *p* = 0.035). Black dots = DG, white inverted triangles = CA, continuous line = DG regression line, dashed line = CA regression line.

For volume analysis, we measured DG and CA separately, and used these areas as a second within-subject variable, in addition to the subregions along the dorso-ventral axis (dorsal, intermediate, and ventral). As expected, CA volume was bigger than DG [$\chi_{\left(1\right)}^{2}$ = 570.661, *p* < 0.001] in all hippocampal subregions. Dorsal areas were larger than intermediate areas and both were larger than ventral areas [$\chi_{\left(1\right)}^{2}$ = 400.586, *p* < 0.001]. Enriched environment had no direct main effect [$\chi_{\left(1\right)}^{2}$ = 0.176, *p* = 0.675], but increased both DG and CA volumes in the dorsal but not in intermediate or ventral subregions [enrichment^*^region $\chi_{\left(2\right)}^{2}$ = 9.825, *p* < 0.007]. The dorsal areas were about 15% larger in the E group than in the C group, while the intermediate and ventral areas were found to be slightly, but not significantly, decreased (15 and 5%, respectively; Figures 8A–C). The relative contribution of DG and CA also differed significantly in the three regions [$\chi_{\left(2\right)}^{2}$ = 582.626, *p* < 0.001], their ratio being smaller in ventral than in dorsal and intermediate subregions (vDG was 21% of vCA, iDG 42% of iCA, and dDG 63% of dCA, respectively). A three-way interaction was found between enrichment, regions and CA vs. DG [$\chi_{\left(2\right)}^{2}$ = 6.159, *p* = 0.046], due to the fact that the magnitude of the enrichment effect differed in different subdivisions.

Using running distance as a co-variate in the GEE (Enriched group only; Figures 8D–F), we found a significant effect of running on hippocampal volumes in both DG and CA [$\chi_{\left(1\right)}^{2}$ = 13.936, *p* < 0.001] and a significant interaction with the three subregions [$\chi_{\left(2\right)}^{2}$ = 84.049, *p* < 0.001]. In particular, both dDG (*r*^2^ = 0.302, *p* = 0.030) and dCA (*r*^2^ = 0.464, *p* = 0.005) volumes were linearly predicted by the distance run. In the rest of the hippocampus, only iCA (*r*^2^ = 0.402, *p* = 0.011) and vCA (*r*^2^ = 0.301, *p* = 0.0343) showed an effect of the distance run.

## Discussion

In this study we showed that 8 days' exposure to an enriched environment, a period much shorter than that usually reported in the literature (Buschler and Manahan-Vaughan, 2012; Ashokan et al., 2016; Koe et al., 2016), differentially affects DCX expression in a dorso-ventral-specific fashion in mice and increases the volume of the dorsal hippocampus. It does not affect *Bdnf* expression, although we did find that voluntary running effort correlated positively with *Bdnf* expression in the ventral DG. Furthermore, we were able to replicate patterns found in immunohistochemistry both with mRNA (RT-PCR; for both DCX and BDNF) and protein (ELISA; for DCX) quantification, using different mice housed in the same cages.

### Response of DCX in dorsal vs. ventral DG

Our treatment had different effects on DCX expression in the dorsal vs. the ventral hippocampus. We found that its expression was upregulated in dDG while it was downregulated in vDG. The fact that the increase in AHN was higher in dDG compared to vDG is in line with other enrichment studies (Tanti et al., 2012, 2013; Ramirez-Rodriguez et al., 2014). The increase in DCX staining in the dDG suggests an increased survival of newly-generated neurons (Rao and Shetty, 2004) and/or an expansion of the dendritic arbor of existing newly-generated neurons. Indeed, voluntary running increases the number of newborn neurons (Van Praag et al., 2000; Kannangara et al., 2009), an effect that is stronger in dDG than in vDG (Nishijima et al., 2013); and environmental enrichment alone is known to increase dendritic complexity of young granule neurons (Beauquis et al., 2010).

However, the decrease in DCX expression in the vDG was unexpected. Our protocol was different from those used in other studies looking at differential regulation of AHN across the dorso-ventral axis of the dentate gyrus in several ways. Firstly, the enrichment (including voluntary exercise) lasted only for 8 days. We know that 8 days of access to running wheels (Jin et al., 2008) and 8 days of exposure to male urine (Mak et al., 2007; Hoffman et al., 2015) both increase AHN, but we do not know if they do this differentially in dDG and vDG. The study that came closest looked after 12 days of voluntary exercise, and found increased AHN in both dorsal and ventral DG (Liu et al., 2013). It is therefore unlikely that the duration of the treatment is responsible for the decreases in DCX expression in the ventral hippocampus.

The one factor that we know to consistently downregulate AHN in the ventral hippocampus is stress. Most protocols looking at the effect of stress on AHN use chronic stressors over many weeks, which decreases survival of progenitor cells in the ventral subregion (Hawley and Leasure, 2012). However, only 4 days of exposure to loud noise can already reduce proliferating cells in the subgranular zone (Gonzalez-Perez et al., 2011), an effect that carried through to a reduction in new DCX^+^ neurons 10 days later. Just 15 min of restraint stress can also reduce the number of proliferating neurons in the subgranular zone (Kannangara et al., 2009). Similarly, short-term experiences of traumatic events (foot shocks inducing learned helplessness) can induce differences in neuronal proliferation and survival as measured by DCX staining that are measurable 8 days after the traumatic event (Ho and Wang, 2010), although in this latter case, most of the effects were in the dorsal, and not the ventral hippocampus. Nevertheless, ventral hippocampus in rats with learned helplessness did also show lower cell proliferation (Ho and Wang, 2010). It is therefore possible that our mice underwent a traumatic experience at the start of the treatment, which reduced the DCX expression in the vDG. One possibility is that the large cages with transparent sides and transparent lids, placed on a table top (instead of cage racks) were stressful to the animals upon initial exposure, and that this reduced the number of DCX expressing cells in the vDG. It is also possible that the exposure to male urine has a different effect in vDG, which was not picked up in the original studies, because they did not differentiate between dDG and vDG (Mak et al., 2007; Hoffman et al., 2015). Further studies would have to be carried out to investigate whether this reduction would be changed over time to a slight increase in DCX, as shown by others after 4–6 weeks of enrichment (Tanti et al., 2012, 2013; Ramirez-Rodriguez et al., 2014), or indeed whether exposure to male urine always reduces DCX expression in the vDG.

### Response of BDNF in dorsal vs. ventral DG

We found no treatment effect on BDNF expression from either immunocytochemistry or RT-PCR in the hippocampus. However, we found that its expression was significantly higher in the vDG compared to the dDG in both treatments and with both detection methods. BDNF is required for the environmental induction of neurogenesis and lack of it can lead to a lack of neurogenic response in a heterozygous knockout mice model (Rossi et al., 2006). However, it is not a stable marker of neurogenesis (Bekinschtein et al., 2007; Callaghan and Kelly, 2012) nor is it only involved in neurogenesis (Nakagawa et al., 2000). It is therefore not surprising to find a difference in the response between BDNF and DCX. Even though BDNF has been shown to respond to voluntary exercise alone (Vaynman et al., 2004; Greenwood et al., 2009) and enriched environment alone is capable of generating a strong BDNF response in dorsal hippocampus (Mosaferi et al., 2015), in some studies, expression of BDNF mRNA in the hippocampus takes longer to induce (Bindu et al., 2007; Kuzumaki et al., 2010). We therefore assume that in our study the treatment was not long enough to trigger the BDNF response.

Interestingly, when we looked at the correlation between BDNF mRNA and the distance run by each mouse, we found a significant correlation. This might indicate that the duration of the experiment was enough to induce mRNA transcription but not long enough for it to be detectable in the protein levels measured by our IHC protocol. The lack of differences in mRNA levels with the control group, which did not have access to running wheels, may lie in the activity patterns of the control animals, which were not measured. Similar to the enriched animals, there may have been individual differences in spontaneous activity (e.g., walking around the cage, climbing on the lids) that caused a wide enough variation in *Bdnf* expression in the control group to overlap with the enriched animals. Alternatively, the individual differences in *Bdnf* observed may predispose animals to running more or less, rather than being a direct consequence of the running. Indeed, in at least one study of forced running (as opposed to voluntary running), BDNF did not relate to intensity or duration of running (Sheikhzadeh et al., 2015), suggesting the causation may go from BDNF to running, and not vice versa.

### Effect of enrichment on hippocampal volume

The enriched environment and the running activity significantly increased both CA and DG volumes in the dorsal hippocampus. In humans also, 12 months of cardiovascular training are associated with an increase in hippocampal volume by about 2% (Erickson et al., 2011). Three main mechanisms could be responsible: the enhancement of capillary density (Black et al., 1990), the generation of new neurons (Van Praag et al., 1999), and increased dendritic branching (Bindu et al., 2007). An increase in gray matter volume is correlated with an increased number of DCX-positive newborn neurons (Biedermann et al., 2016), suggesting that the increased DCX signal we found in dDG may be related to the increased volume found in the dorsal hippocampus.

EE significantly increases the DG volume (Llorens-Martín et al., 2007) and the changes in DG volume might be related to increases in the size of the dendritic trees of granule cells as a result of alterations in the animal's environment (Faherty et al., 2003). Therefore, our results suggest that with only 8 days of EE we are able to trigger a regional enhancement of dendritic branching in the dorsal hippocampus, resulting in an increased volume. Indeed, increased branching has been demonstrated before after only 10 days of enrichment (Bindu et al., 2007). The increase in DCX expression in the dorsal DG could be a combination of an increase in dendritic branching in the maturing neurons, and more new neurons. The DCX and volume results may therefore both reflect increased dendritic branching in the dorsal hippocampus, which we did not measure directly. Volumes of hippocampal subdivisions were significantly related to the distance the animals had run during the experiment. This effect was strongest in the dorsal hippocampus, which is also the region that was most affected by the enrichment. This suggests that in this case, it is likely that voluntary exercise has influenced dorsal hippocampal volume.

### Methodological implications

In our study, quantification of mRNA and protein gave us comparable results to what we found using more traditional immunohistochemistry combined with image analysis. This is consistent with another study (Kremer et al., 2013) in which running-induced changes in Dcx protein and mRNA levels followed those measured using immunohistochemistry. In that study, it was clear that baseline levels of DCX mRNA and protein are present that are undetectable using IHC. In our experiment as well, baseline patterns of DCX expression along the dorso-ventral axis differed among the different techniques, but the induced changes were consistent. This was different in our analysis of BDNF, which showed the same dorsoventral pattern in both IHC and RT-PCR. Although often a weak correlation is found between mRNA and protein abundances (Maier et al., 2009), differentially expressed mRNAs correlate significantly better with their protein product than non-differentially expressed mRNAs (Koussounadis et al., 2015) and that may be the case in both our and Kremer et al.'s studies when neurogenesis is being manipulated by environmental conditions.

Because amounts of dissected tissue and technical factors such as RNA or protein extraction and reverse transcription can differ in their efficiency from sample to sample, it is important to normalize expression to a “housekeeping gene.” Because changes in DCX represent changes of its expression in new dentate gyrus granule cells, we normalized our DCX expression for both mRNA and protein content to a protein that is uniquely expressed in all granule cells from the moment of cell fate specification throughout their lives: PROX1 (Karalay et al., 2011; Stergiopoulos et al., 2014). This is analogous to our anatomical quantification, in which we expressed DCX as a proportion of the total surface of the GCL. Using PROX1 also allows us to control for the specificity of the dissection technique and consequently decrease the variation due to accidental inclusion of tissue not belonging to the DG. The normalization over PROX1 allowed us to be more specific in quantifying DCX expression in granule cells and to more easily relate the three different measurements with each other. Molecular methods are less time consuming, and therefore more suitable for the use in higher throughput applications in the study of AHN, possibly including studies of pathologies in which AHN plays a crucial role (e.g., depression, Alzheimer's disease). The drawback is that the tissue is homogenized. It is therefore important to dissect the functionally different parts of the DG away from each other, as they might respond differently to the treatment. If we had combined dorsal and ventral hippocampus in one sample, the differential response in the two subdivisions would have canceled each other out.

## Conclusions

In the present study we have shown that a short enrichment paradigm can trigger an altered neurogenic response in hippocampal DG. DCX expression was increased in dDG and decreased in vDG. In dDG, the increase in DCX is associated with an increase in the volume of the entire dorsal hippocampus. The decreased DCX expression in vDG in the enriched group was unexpected. We have speculated that maybe the animals experienced stress upon introduction to the new environment, or alternatively, that the addition of male urine to the cages affected vDG differently from dDG. Only further studies can determine which is the correct interpretation. Outcomes from alternative ways of measuring DCX (from mice housed in the same cage) are consistent with each other, indicating that for DCX induction or reduction, more rapid quantification methods than immunohistochemistry could be used. This is relevant in settings where quicker molecular quantification of neurogenesis may be advantageous such as in studies of disease.

## Ethics statement

This study was carried out in accordance with the recommendations of the Guidelines for the Use of Animals, published by the Association for the Study of Animal Behavior. The protocol was approved by the Animal Welfare and Ethical Review Board of Newcastle University (approval #343). All animals were killed using Schedule 1 methods, as required by the regulations set out by the Animals in Scientific Procedures (1986) Act of the United Kingdom.

## Author contributions

FG, TB, and TS designed the study and drafted the manuscript; EA and NW estimated the physical activity; FG and GL performed immunohistochemistry; TM estimated hippocampal volume, FG performed RT-PCR; CB and RG performed ELISA; FG and TS performed statistical analysis. All authors read and approved the final manuscript.

### Conflict of interest statement

The authors declare that the research was conducted in the absence of any commercial or financial relationships that could be construed as a potential conflict of interest.

## Supplementary material

Figure S1 DG regions identification. To transfer the “X shaped structure” of pyramidal neuron belonging to CA3i (Fanselow and Dong, 2010) that denotes the iDG, we used images from the software Brain Explorer 2 (Allen Brain Institute, US). We initially define this region **(A)** and translate it from the sagittal to coronal plane **(B)**. We then used these landmarks throughout the all coronal plane **(C–F)** and representative acquired images are marked with an apostrophe **(C′–F′)**.

Figure S2 Thresholding method for DCX image quantification. Images acquired with 20X objective magnification were processed with ImageJ software and the GCL region **(A)** was outlined (black line) in all the analyzed frames. Images were acquired with the same amount of light and intra animal variability was overridden by setting the threshold level manually for each slide. The initial CYMK image **(C_1_)** was converted in the 8-bit image and on the cut GCL **(C_2_)** the threshold was applied. The green triangle **(C_3_)** represents a region within the GCL not presenting DCX signal so it was used as minimum gray value. The software's histogram function **(C_4_)** gave us Max, Min, Mean, and *SD* gray scale values for each image in the 8 bit gray scale (0 < gray < 256). From the Mean value found in the histogram we subtracted two *SD* (Threshold = Mean – 2SD) and we used that value as our minimum gray. All the composite images were elaborated in the same way and the threshold applied to every frame in a montage (**B**, sequential 1–8).
