# Supplementary figures and images for: Effects of Environmental Enrichment on Doublecortin and BDNF Expression along the Dorso-Ventral Axis of the Dentate Gyrus
Source: Front Neurosci. 2017 Sep 15;11:488. doi: 10.3389/fnins.2017.00488 (PMC5605570; doi:10.3389/fnins.2017.00488)

E'

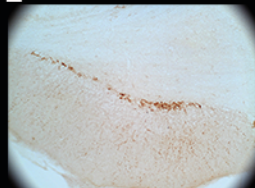

D'

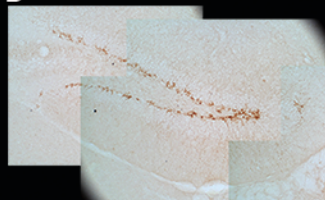

C'

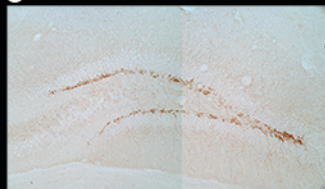

F'

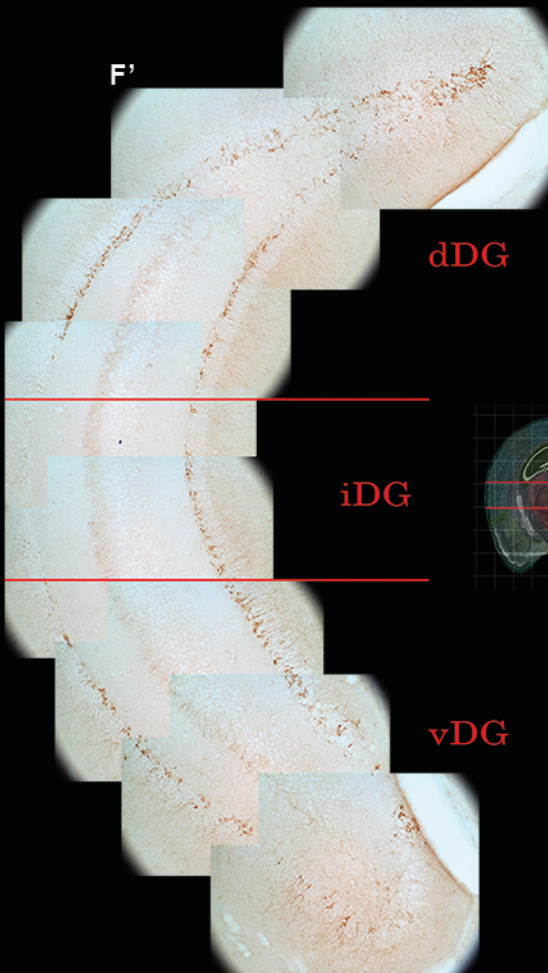

dDG

iDG

vDG

A

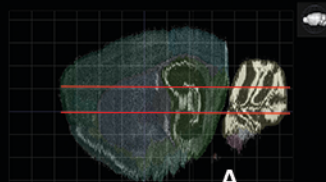

B

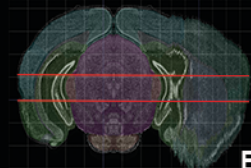

C

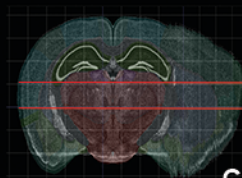

D

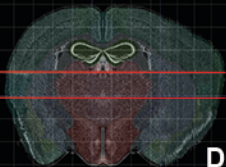

E

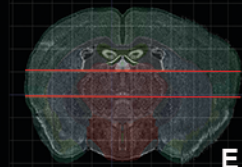

F

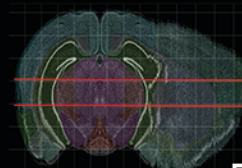

Supplement: Figure S1 — DG regions identification. To transfer the “X shaped structure” of pyramidal neuron belonging to CA3i (Fanselow and Dong, 2010) that denotes the iDG, we used images from the software Brain Explorer 2 (Allen Brain Institute, US). We initially define this region (A) and translate it from the sagittal to coronal plane (B). We then used these landmarks throughout the all coronal plane (C–F) and representative acquired images are marked with an apostrophe (C′–F′). [file Image1.pdf]

A

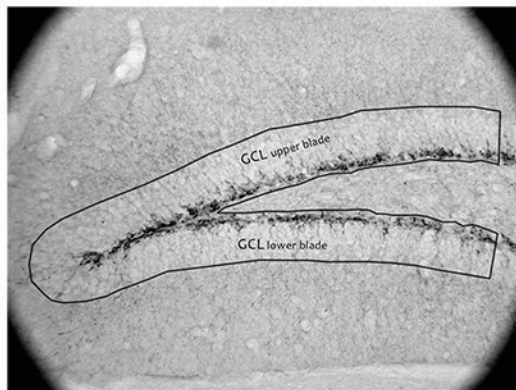C<sub>1</sub>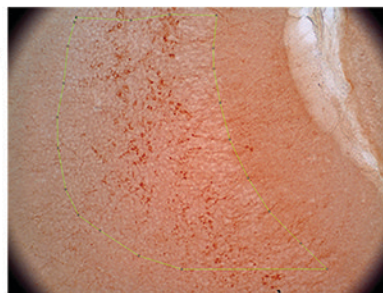C<sub>2</sub>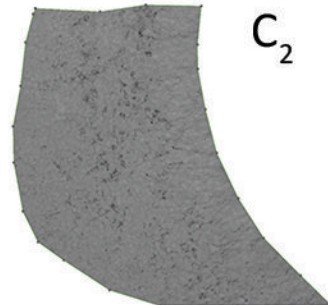

B

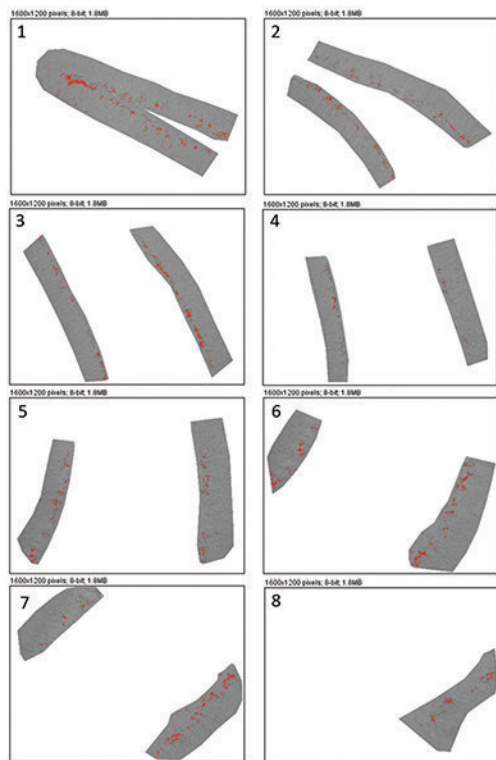C<sub>3</sub>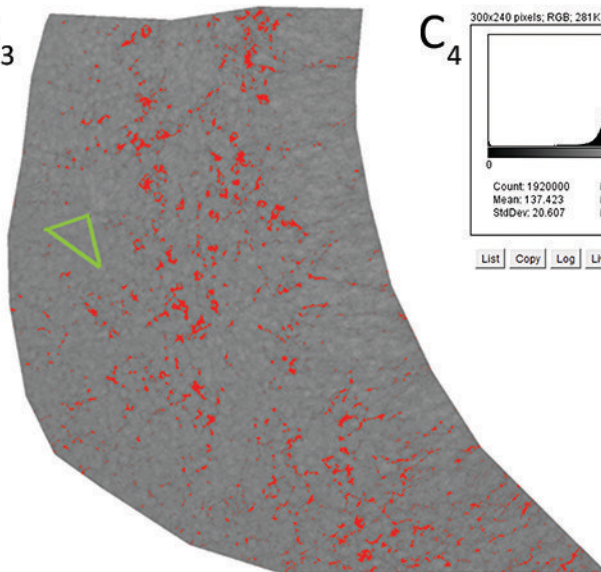C<sub>4</sub>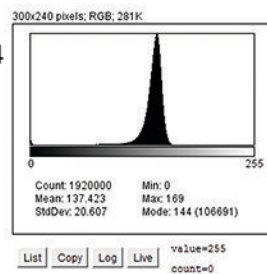

Supplement: Figure S2 — Thresholding method for DCX image quantification. Images acquired with 20X objective magnification were processed with ImageJ software and the GCL region (A) was outlined (black line) in all the analyzed frames. Images were acquired with the same amount of light and intra animal variability was overridden by setting the threshold level manually for each slide. The initial CYMK image (C1) was converted in the 8-bit image and on the cut GCL (C2) the threshold was applied. The green triangle (C3) represents a region within the GCL not presenting DCX signal so it was used as minimum gray value. The software's histogram function (C4) gave us Max, Min, Mean, and SD gray scale values for each image in the 8 bit gray scale (0 < gray < 256). From the Mean value found in the histogram we subtracted two SD (Threshold = Mean – 2SD) and we used that value as our minimum gray. All the composite images were elaborated in the same way and the threshold applied to every frame in a montage (B, sequential 1–8). [file Image2.pdf]
